# Cadmium and lithium doping in silver orthophosphate: An *ab initio* study

**DOI:** 10.1038/srep32574

**Published:** 2016-08-31

**Authors:** Yang Huang, Ming-Yang Liu, Tai Ma, Zhong-Ping Lou, Chao Cao, Yao He

**Affiliations:** 1 Department of Physics, Yunnan University, Kunming 650091, China; 2 Department of Physics, Hangzhou Normal University, Hangzhou 310036, China

## Abstract

Using hybrid functional calculations, we investigate the effects of defects and defect complexes related with Cd, Li, and N impurities on the atomic and electronic properties of Ag_3_PO_4_. It was found that substitutional Cd on Ag lattice site (Cd_Ag_) contributes to the *n*-type conductivity of Ag_3_PO_4_. For substitutional Cd on P (or O) lattice site (Cd_P_) (or Cd_O_), it is not expected that Cd will incorporate into the P (or O) site due to the strong covalent interactions in the PO_4_ structural units. The interstitial Cd (Cd_i_) acts as a shallow donor, but its formation energy is relatively high compared with that of Cd_Ag_. For the (Cd_Ag_-2N_O_) complex, the formation of this inactive complex generates a fully occupied impurity band just above the valence band maximum of Ag_3_PO_4_, which significantly reduces the acceptor transition energy level. But the formation energy of the (Cd_Ag_-2N_O_) complex is even higher than that of the corresponding single point defect N_O_. Unlike Li_P_ and Li_O_ which has relatively high formation energy, interstitial Li (Li_i_ or Li_i_(s)) with an appreciable solubility is likely to be the *n*-type dopant under O-poor condition.

Owing to that fact that oxide semiconductor photocatalysts have promising applications in the development of renewable energy (e.g., converting solar energy into chemical fuels) and the treatment of environment pollution (e.g., degradation of pollutants), much attention has been paid to these materials over the past several decades^1^,^2^,^3^. One of the most popular oxide semiconductors for this purpose, titanium dioxide (TiO_2_), based on its high photocatalytic activity, resistance to photocorrosion, low cost and non-toxicity, has received intense research interest as a promising photocatalyst for water splitting and hydrogen production^4^,^5^. However, the intrinsic wide band gap of pure TiO_2_ (~3.2 eV for anatase and ~3.0 eV for rutile) confines its photon absorption to the ultraviolet (UV) region, severely limiting solar energy utilization to ~5%. Hence great efforts have been made to extend the photoabsorption of TiO_2_ to the visible light region^5^,^6^,^7^. Recently, silver-based oxides semiconductors are reported to own high photocatalytic activation in the visible light region, such as Ag_3_VO_4_^8^, AgGaO_2_^9^, AgSbO_3_^10^. Especially, Ye *et al*.^11^ have found that Ag_3_PO_4_ exhibits strong oxidation power leading to O_2_ production from water, and the quantum yield achieve up to nearly 90% under visible light. This is intriguing because most photocatalysts give much poorer quantum yields of ~20%^12^. Therefore, further studies^13^,^14^,^15^,^16^,^17^ were carried out to understand the mechanism of its high water-oxidation activity. For example, Umezawa *et al*.^18^ revealed that the excellent photocatalytic performance of Ag_3_PO_4_ is attributed to the Ag *s*-*s* hybridization without localized *d* states which results in the highly dispersive band structure of the CBM of Ag_3_PO_4_. Although pure Ag_3_PO_4_ achieves an extremely high quantum yield under visible light, it has been recently found that native point defects are unlikely responsible for the electrical conductivity^12^. However, photoelectrochemical cell needs two-electrode to convert light energy into electric energy, e.g., metal cathode and photoanode made of *n*-type semiconductor^16^,^19^. Therefore, we expect that external doping can further improve the photocatalytic activity of this novel photooxidation material.

The valence-band maximum (VBM) of Ag_3_PO_4_ composes of mostly Ag 4*d* and O 2*p* states^20^. Oxygen is much more electronegative than the attempted nitrogen acceptor dopant, and the transition energy level of the N_O_ acceptor should be high. Indeed, the calculated transition level for single acceptor N_O_ is 0.4 eV above the VBM of Ag_3_PO_4_^17^. On the other hand, since the cohesive energy of N_2_ is large, the chemical potential of N_2_ is very low and the formation energy of N_O_ is very high^21^. To obtain net *p*-type Ag_3_PO_4_, the low solubility of N monodoping is unlikely to generate sufficient number of holes to compensate the free electrons. Successful *p*-type doping requires a shallow delocalized acceptor level. But doping generally destroys the local symmetry, and the chemical mismatch between the host and dopant may result in deep defect levels in the band gap^22^. The codoping method was originally proposed by Yamamoto and Katayama-Yoshida for carrier control in wide-band-gap semiconductors^23^. Using codoping method one may be able to enhance the dopant solubility and lower the defect transition energy levels. However, further study found that although codoping could be useful in increasing dopant incorporation, the different wave function characters and symmetry of donor and acceptor levels may result in a too weak repulsion between these levels, and codoping will fail to reduce the defect transition energy levels^24^,^25^. In order to decrease the ionization energy of N acceptor in ZnO, Yan *et al*.^25^ proposed the impurity band model to explain the real codoping mechanism. The essence of this model is the rise of the VBM. According to the “doping pinning rule”, the lower the VBM of a semiconductor is, the higher the probability that the semiconductor cannot be doped *p*-type is^26^. Moreover, Li *et al*.^27^ considered that the anion and cation kinetic *p-d* repulsion was one of the reasons why N_O_ defect level is deep in ZnO, if replacing Zn atom by isovalent Mg or Be atom containing no *d* orbital, the defect transition energy level of nX_Zn_-N_O_ (X = Mg, Be, n = 1, 2, 3, 4) should be lower than that of N_O_ in ZnO. But there is little studies on Li (without occupied *d* orbital) codoping in Ag_3_PO_4_, which will be addressed in this work.

In this present work, we used screened Coulomb potential hybrid DFT calculations to investigate the atomic structure and electronic properties of Cd, Li monodoping and (Cd, N), (Li, N) codoping in Ag_3_PO_4_. We calculate the formation energies and the transition energy levels of Cd_Ag_, Cd_P_, Cd_O_, Cd_i_, Cd_i_(s), the (Cd_Ag_-2N_O_) complex, Li_P_, Li_O_, Li_i_, Li_i_(s), and the (Li_Ag_-N_O_) complex, as well as the binding energies of all defect complexes. The results show that Cd_Ag_ and Li_i_ (or Li_i_(s)) may serve as possible sources of *n*-type conductivity under O-poor conditions. Cd_Ag_ is likely to bind with N_O_ to form the (Cd_Ag_-N_O_) complex with a binding energy of −2.02 eV, and doping further N atom results in the (Cd_Ag_-2N_O_) complex, which acts as acceptor with a significantly smaller transition level compared to the isolated N acceptor in Ag_3_PO_4_.

## Methods

Our calculations are based on DFT^28^ and projector-augmented wave potentials^29^ as implemented in the Vienna *ab initio* simulation package (VASP) code^30^. The exchange correlation potential is treated with the screened hybrid functional of Heyd, Scuseria, and Ernzerhof (HSE)^31^. We find that a proportion of 33% HF exchange with 67% PBE exchange produces accurate values for lattice constants and the band gap in Ag_3_PO_4_. The electron wave function was expanded in plane waves up to a cutoff energy of 300 eV and a Monkhorst-Pack *k*-point mesh^32^ of 2 × 2 × 2 was used for geometry optimization and electronic structure calculations. Both the atomic positions and cell parameters were optimized until residual forces were below 0.01 eV/Å. The optimized cell parameters are *a* = *b* = *c* = 6.02 Å, in excellent agreement with the experimental values of *a* = *b* = *c* = 6.00 Å^20^. The calculated indirect band gap (M−Γ) and the direct band gap (Γ) are 2.33 eV and 2.45 eV, in excellent agreement with the experimental value of 2.36 and 2.43 eV, respectively^11^. A 128-atom supercell is used to simulate Cd, Li monodoping and (Cd, N), (Li, N) codoping in Ag_3_PO_4_.

The likelihood of incorporating an impurity in Ag_3_PO_4_ is determined by its formation energy. In this paper, the formation energy of a charged defect is defined as the following:^33^


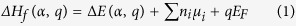



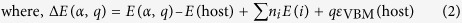


A more detailed discussion of the formation energy is described elsewhere^34^,^35^. When Ag_3_PO_4_ is grown under thermal equilibrium conditions, the chemical potentials of the constituent atoms, which is referenced to the values of their elemental forms, must satisfy the equilibrium condition 3*μ*_Ag_ + *μ*_P_ + 4*μ*_O_ = Δ*H*_*f*_ (Ag_3_PO_4_) = −10.46 eV, where Δ*H*_*f*_ (Ag_3_PO_4_) is the formation enthalpy of Ag_3_PO_4_. In addition, we must enforce the constraint such as 2*μ*_Ag_ + *μ*_O_ < Δ*H*_*f*_ (Ag_2_O), to prevent the precipitation of Ag_2_O during the growing process of Ag_3_PO_4_. Similar constraint must be applied to P_2_O_5_^17^. To avoid the precipitation of constituent atoms, *μ*_Ag_, *μ*_P_, *μ*_O_ must satisfy *μ*_Ag_ < 0, *μ*_P_ < 0, *μ*_O_ < 0, respectively. For impurity doping, the chemical potentials of impurities also need to satisfy other constraints to avoid the formation of impurities-related phases. Specifically, the chemical potential of Cd is constrained by *μ*_Cd_ < 0 and *μ*_Cd_ + *μ*_O_ < Δ*H*_*f*_ (CdO) = −2.59 eV (experimental value^36^: −2.68 eV and theoretical value^36^: −2.40 eV). Also for Li doping, the chemical potential of Li is constrained by *μ*_Li_ < 0 and 2*μ*_Li_ + *μ*_O_ < Δ*H*_*f*_ (Li_2_O) = −5.96 eV (experimental value^37^: −6.20 eV and theoretical value^38^: −6.28 eV). We take *μ*_N_ with respect to the energy per atom of N_2_ molecule.

The binding energy of a complex defect represents energy changing from isolated dopants to a complex defect. To determine whether the binding of two dopants is energetically preferred (e.g., Cd_Ag_ and N_O_), we calculate the binding energy *E*_b_, which is defined as^39^,^40^,^41^


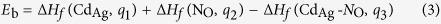


where *q*_*1*_, *q*_*2*_, and *q*_*3*_ are the most stable charge states of Cd_Ag_, N_O_, and complex (Cd_Ag_-N_O_), respectively, at any given *E*_F_. A positive *E*_b_ indicates the reaction is exothermic and the defect pair tends to bind to each other when both are present in the system.

## Results and Discussion

### Cd monodoping

Cadmium sits to the right of Ag in the periodic table, having an atomic size very close to that of Ag which makes sure that negligible strain energy is introduced to the Ag_3_PO_4_ host. Thus, we expect Cd to preferentially occupy the Ag site in Ag_3_PO_4_, leading to relatively small relaxations and acting as a shallow donor. Indeed, in the neutral charge state (Cd_Ag_^0^), the neighboring O atoms relax inward, resulting in a Cd-O bond length (2.21 Å) that is 6.8% shorter than the equilibrium Ag-O bond length (2.37 Å), while the Cd-Ag bond length (3.22 Å) becomes 7% longer than the equilibrium Ag-Ag bond length (3.01 Å). In the positive charge state (Cd_Ag_^+1^), the Cd-O bond length is 2.2 Å, and the Cd-Ag bond length is 3.23 Å. It is noteworthy that the Cd-O bond length is similar to the counterpart (2.35 Å) in CdO unit cell^42^.

**Figure 1:**
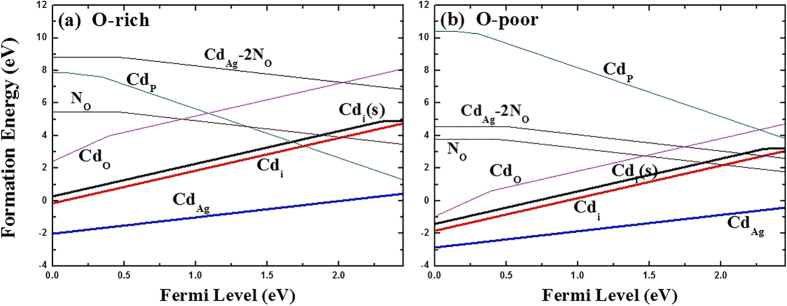
The formation energies of possible Cd monodoped defects, Cd_Ag_, Cd_P_, Cd_O_, Cd_i_ and Cd_i_(s), together with (Cd_Ag_-2N_O_) complex as a function of the Fermi energy under O-rich (**a**) and O-poor (**b**) conditions. Only the lowest formation energy states are shown.

As expected, Cd_Ag_ is shallow donor, and the transition energy level of *ε*(0/+) is located at 0.01 eV below the CBM. The conductivity of a semiconductor depends not only on the transition levels of donor or acceptors, but also on their formation energies. The results of formation energy for the defects under consideration are listed in Table 1. Figure 1 shows the calculated formation energies of Cd_Ag_ as a function of Fermi level under O-rich [Fig. 1(a)] and O-poor [Fig. 1(b)] conditions, respectively. Under O-poor growth condition [Fig. 1(b)], the formation energy of Cd_Ag_ is much lower than that under O-rich condition, and becomes negative in the entire range of *E*_F_ value in the band gap. This is because the chemical potential of Cd is limited by the O chemical potential due to the limit of impurity-related phase CdO. Therefore, the highest possible *μ*_Cd_ requires the lowest *μ*_O_, i.e. O-poor condition. Our results suggest that *n*-type Ag_3_PO_4_ is achievable by Cd-doping under O-poor condition.

When Cd dopant occupies the P lattice site, this defect behaves as a triple acceptor. The transition energy levels of *ε*(−/0), *ε*(2−/0) and *ε*(3−/0) are located at 0.13 eV, 0.27 eV and 0.25 eV above the VBM, respectively. In general, deep impurity-related levels typically represent localized electron distributions, and a change of occupancy usually results in change of structures^43^. Indeed, because of the large size mismatch between the Cd and P atoms, the four oxygen neighbors of Cd_P_ lie at a distance of 2.20–2.21 Å, which are larger than the equilibrium P-O bond length of 1.56 Å for bulk Ag_3_PO_4_. As seen in Fig. 1, the formation energy of Cd_P_ is quite high even under extreme O-rich condition. Moreover, recent studies, based on a revised molecular orbital diagram for Ag_3_PO_4_, have pointed out that the strong covalent interactions are formed in the tetrahedral PO_4_ structural units^14^. It is therefore not expected that Cd will incorporate on the P site with an appreciable solubility.

Since oxygen atom can provide six electrons and cadmium can provide only two electrons, it is expected that Cd_O_ acts as donor. Indeed, the calculated transition energy levels of *ε*(0/+), *ε*(0/2+), *ε*(0/3+) and *ε*(0/4+) are located at −0.02 eV, −0.24 eV, 0.53 eV and 0.91 eV below the CBM, respectively. For Cd_O_, in the 0, 1+, 2+, 3+ and 4+ charge states, the distances between Cd and the nearest-neighbor O atoms are 3.32, 3.05, 2.88, 2.86 and 2.84 Å, respectively. This dramatic lattice distortion can lead to higher formation energy. According to Fig. 1, Cd_O_ has much lower formation energy under O poor condition than under O rich condition. But even under O poor condition, Cd_O_ has a formation energy that is 1.91 eV higher than that of Cd_Ag_ at the VBM, and so its contribution is less significant.

**Figure 2:**
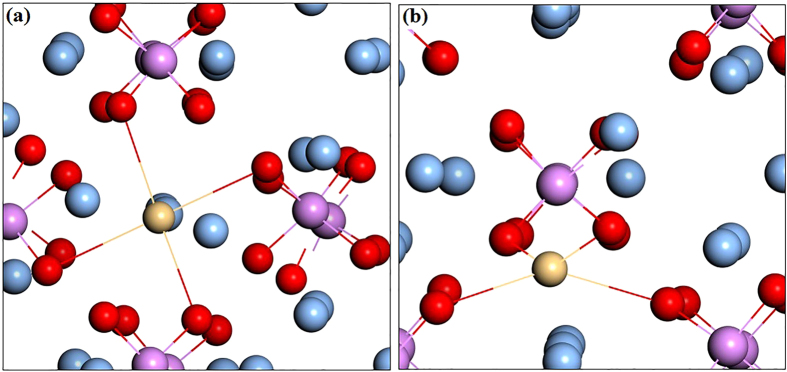
Atomic geometry of the relaxed structures of the (**a**) Cd_i_ and (**b**) Cd_i_(s) configurations in Ag_3_PO_4_.

For interstitial Cd, we have studied two possible atomic configurations. One is the tetrahedral site coordinated by four O atoms (expressed as Cd_i_), the other is the so-called split interstitial site which consists of two atoms on a single substitutional lattice site (expressed as Cd_i_(s)), as shown in Fig. 2. For Cd_i_(s), this configuration is 0.16 eV higher in energy than Cd_i_, and the calculated transition energy levels of *ε*(0/+) and *ε*(0/2+) are located at 0.27 eV and 0.14 eV below the CBM, which are 0.17 eV and 0.13 eV higher than that of Cd_i_, respectively. Therefore, in the following discussion we consider only the Cd_i_ configuration. In the neutral charge state, the distance between Cd and the nearest-neighbor O atom is 2.33 Å which is very close to the Cd-O bond length in CdO unit cell. In the 1+ and 2+ charge states, the Cd-O bond lengths are 2.29 and 2.26 Å, respectively. As shown in Fig. 1, the calculated formation energies of Cd_i_^2+^ are −0.14 and −1.83 eV under O-rich and O-poor conditions at the VBM, respectively. It implies that O-poor condition would produce much more abundant numbers of Cd donors in comparison with O-rich condition. However, its formation energy is relatively high compared with that of Cd_Ag_ under both O-rich and O-poor conditions, regardless of the Fermi level position. Our results indicate that Cd_i_ will not be a relevant configuration for Cadmium in Ag_3_PO_4_.

### Cd codoping

**Figure 3:**
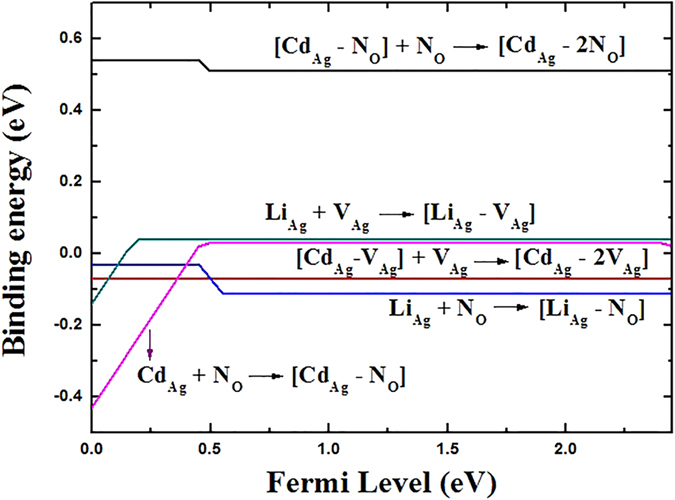
Binding energies *E*_b_ of complex defects as a function of the Fermi level.

**Figure 4:**
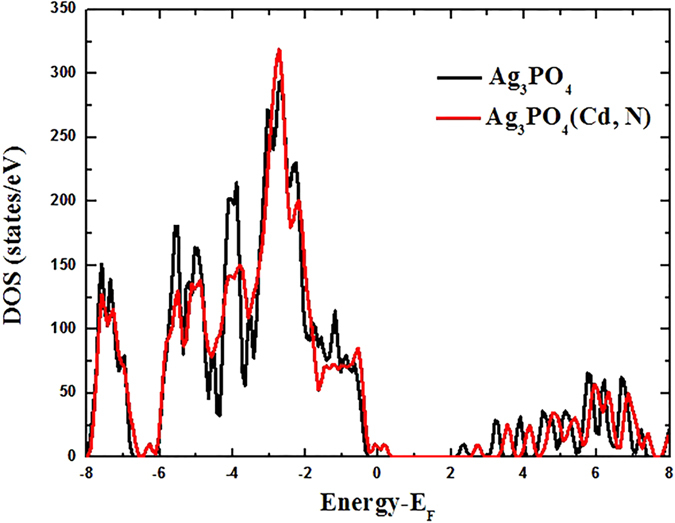
The calculated total DOS for pure Ag_3_PO_4_ and a supercell containing a (Cd_Ag_-N_O_) complex.

The formation of the (Cd_Ag_-N_O_) complex is a result of Coulomb binding between positively charged donor (Cd_Ag_) and negatively charged acceptor (N_O_). To see if the complex can form, we calculated the binding energy. We find that Cd_Ag_ is likely to bind with N_O_ to form the (Cd_Ag_-N_O_) complex with a binding energy of 0.03 eV. The positive binding energy means that the complex is energetically favorable and can be seen as the passive complex. Obviously, the (Cd_Ag_-N_O_) complex is dielectric and will not provide electron or hole in Ag_3_PO_4_. But it may affect the electrical properties by changing the electronic structure^44^. The values of *E*_b_ for this complex are shown in Fig. 3 as a function of the Fermi level. Figure 4 shows the calculated total DOS of Ag_3_PO_4_ with and without the (Cd_Ag_-N_O_) complex in order to see the effect of passivation on relative shift of the VBM. As can be seen from Fig. 4, the formation of a passive (Cd_Ag_-N_O_) complex does not change the basic electronic structure, but only generates an additional fully occupied impurity band with the energy width of 0.54 eV above the VBM of Ag_3_PO_4_. The distance between Cd and N in the complex is 2.14 Å. The bond length of Cd and its nearest-neighbor O atom is 2.18 Å, which is 8% shorter than the equilibrium Ag-O bond length. The Cd-Ag bond length (3.01 Å) becomes 2% longer than the equilibrium Ag-Ag bond length.

**Figure 5:**
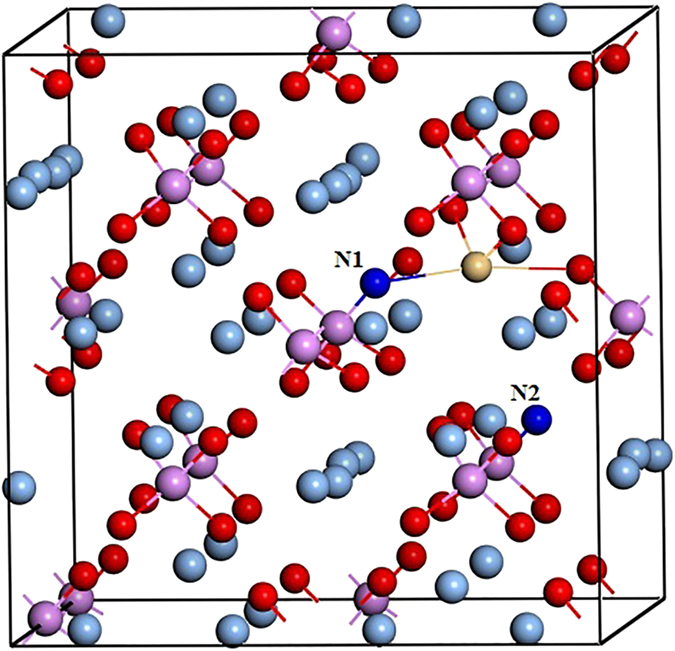
Crystal structure of Ag_3_PO_4_ supercell with codoping of Cd_Ag_ and two N_O_. The silver, pink, red, khaki and blue balls represent Ag, P, O, Cd and N atoms, respectively.

When a second N atom is added to a neighbor site of the passive (Cd_Ag_-N_O_) complex (the configuration of Cd_Ag_-2N_O_ complex is shown in Fig. 5), we find that the binding energy of (Cd_Ag_-2N_O_) is 0.51 eV with respect to the (Cd_Ag_-N_O_) complex and N_O_. The positive binding energy means that the *p*-type complex is energetically stable and can form if more acceptors are doped into Ag_3_PO_4_. Based on the fact that the transition will occur between the N defect levels and the fully occupied impurity bands rather than the original valence band^25^, we calculated the transition energy level *ε*(−/0) of (Cd_Ag_-2N_O_) which is 0.49 eV above the VBM of Ag_3_PO_4_. And the value is reduced to −0.05 eV from the impurity band of the passive (Cd_Ag_-N_O_) complex. Therefore, the transition level can be reduced dramatically. In the neutral charge state (Cd_Ag_-2N_O_)^0^, the distances between Cd and N1, Cd and N2 atoms in the complex are 2.16 and 4.09 Å, respectively. The bond lengths of Cd-O and N1-P are 2.18 and 1.65 Å, which are 8% shorter and 5.8% longer than the Ag-O and O-P bond lengths in the bulk, respectively. The distance between Cd and Ag is 3.12 Å along the b axis, which is 3.7% longer than the corresponding bond length of Ag-Ag in bulk Ag_3_PO_4_. Whether a dopant can be a good acceptor, it should have high solubility under proper conditions, except low transition energy. Figure 1 has depicted the calculated formation energies as a function of Fermi level for (Cd_Ag_-2N_O_) under O-rich and O-poor conditions, respectively. We can see that the formation energy of the (Cd_Ag_-2N_O_) acceptor is relatively high even under O-poor condition. If the formation energy is higher than that of the corresponding single points defects Cd_Ag_ and N_O_^17^, it is because the energy cost to create the extra single point defect is larger than the Coulomb interaction between the donors and acceptors^24^. Thus, *p*-type conductivity would not be readily achieved by (Cd, N) codoping of Ag_3_PO_4_.

From what has been discussed above, Cd_Ag_ is suggested to be the dominant donor in Cd-doped Ag_3_PO_4_. Combined with the fact that “electron killer” is Ag vacancy (V_Ag_)^16^, the natural candidate for a possible complex is the Cd_Ag_ donor and V_Ag_ acceptor pair. Indeed, our hybrid functional calculations indicate that the (Cd_Ag_-2V_Ag_) complex is an acceptor with the transition level *ε*(−/0) of 0.18 eV above the VBM. In order to increase the distance between two V_Ag_ and weaken the level repulsion between them, we have established a configuration that two V_Ag_ acceptors are connected by Cd_Ag_ donor, as proposed by Limpijumnong *et al*.^45^ in ZnO (As_Zn_-2V_Zn_). However, the binding energy for the (Cd_Ag_-2V_Ag_) complex is negative at −0.08 eV. It implies that this kind of defect complex is unlikely to exist in Ag_3_PO_4_.

### Li monodoping

**Figure 6:**
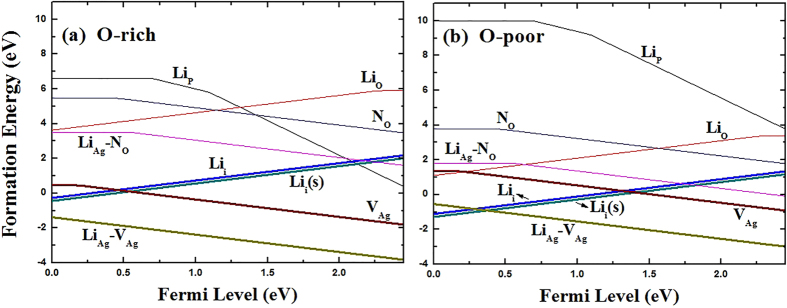
The formation energies of possible Li monodoped defects, Li_P_, Li_O_, Li_i_ and Li_i_(s), together with (Li_Ag_-N_O_) complex and (Li_Ag_-V_Ag_) complex as a function of the Fermi energy under O-rich (**a**) and O-poor (**b**) conditions. Only the lowest formation energy states are shown.

Since Li and Ag are isovalent, Ag substituted by Li (Li_Ag_) is electrically inactive and Li monodoping is not considered. Similar to Cd_P_, Li_P_ has rather high formation energies under both O-rich and O-poor conditions (as shown in Fig. 6) because of the strong covalent interaction in the PO_4_ units, which indicates that the formation of Li_P_ is energetically unfavorable. The calculated results are summarized in Table 1. In the neutral charge state, the four equivalent oxygen atoms move outward by 25% of the initial P-O bond length. For Li substituting O (Li_O_), the calculated formation energies of Li_O_ are lower than Li_P_, mainly because of the small misfit of atomic radius. The distance between Li and the nearest-neighbor O atom is 1.9 Å in the neutral charge state, and this highly perturbed structure can be described as Li going to an interstitial site and leaving behind an O vacancy^43^. The calculated transition energy levels *ε*(0/+), *ε*(0/2+) and *ε*(0/3+) are located at 0.18 eV, 1.55 eV and 2.0 eV below the CBM, respectively.

**Figure 7:**
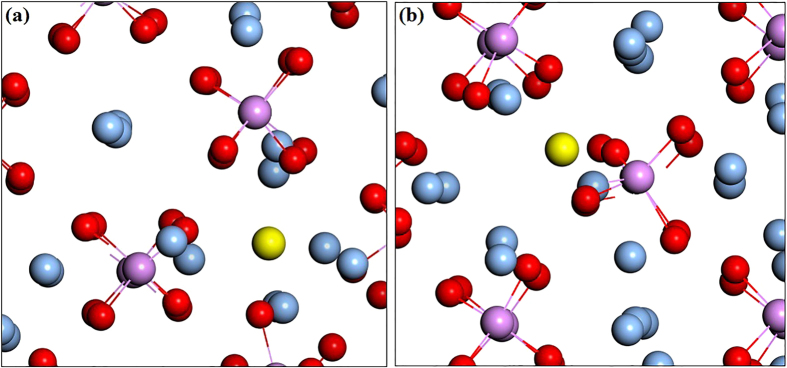
Atomic geometry of the relaxed structures of the (**a**) Li_i_ and (**b**) Li_i_(s) configurations in Ag_3_PO_4_.

Although substitutional Li behaves as an acceptor, Li tends to occupy the interstitial site and loose its outmost electron to behave as a donor. This will lead to the self-compensation and limit its application as an efficient *p*-type dopant in ZnO^27^. Therefore, we have also studied two possible atomic configurations for interstitial Li as shown in Fig. 7. The most stable configuration is the split interstitial doping and Li_i_(s) is only 0.06 eV lower in energy than Li_i_. The calculated transition energy level *ε*(0/+) for Li_i_ and Li_i_(s) are found to lie at 0.04 and 0.15 eV above the CBM, respectively. Figure 6 shows the calculated defect formation energy as a function of the Fermi level under O-rich and O-poor conditions. From Fig. 6, it was found that Li impurity prefers interstitial sites over substitutional sites regardless of the Fermi level position. For example, the formation energy of Li_i_ (Li_i_(s)) is 2.21 eV (2.38 eV) lower than that of Li_O_ under O-poor condition. However, the formation energies of the positively charged interstitial Li (Li_i_ or Li_i_(s)) increase with the Fermi energy while the negatively charged complex (Li_Ag_-V_Ag_) decrease with the Fermi energy and the (Li_Ag_-V_Ag_) complex is not stable (see discussion later). Our analysis suggests that interstitial Li (Li_i_ or Li_i_(s)) is likely to be the *n*-type dopant under O-poor condition.

### Li codoping

Because Li_Ag_ is electrically inactive and N_O_ is a deep acceptor, the (Li_Ag_-N_O_) complex is an acceptor. The calculated transition energy level *ε*(−/0) for the complex is located at 0.54 eV above the VBM, deeper than the corresponding single N dopant (0.46 eV)^17^. It is because the radius of Li is very different from Ag. To make sure whether the complex can form, we have also calculated the binding energy. The calculated binding energy for the (Li_Ag_-N_O_) complex is negative (−0.11 eV), indicating that Li_Ag_ will not bind with N_O_ in Ag_3_PO_4_. This is likely because the Ag-N bond is stronger than the Li-N bond. According to our calculations, the Ag-N bond length (2.11 Å) is shorter than the Li-N bond length (4.61 Å). It should be that the stable *p*-type conductivity will not be achieved by the (Li_Ag_-N_O_) complex. We also calculated the transition energy level *ε*(−/0) for the (Li_Ag_-V_Ag_) complex, which is located at 0.04 eV below the VBM. The binding energy of this complex is positive (0.04 eV), which implies that this complex is stable in Ag_3_PO_4_.

## Conclusions

In summary, we have investigated the formation of isolated defects and defect complexes in Cd and Li monodoped, (Cd, N) and (Li, N) codoped Ag_3_PO_4_ by the hybrid functional calculations. It is found that Cd_Ag_ contributes to the *n*-type conductivity of Ag_3_PO_4_ under O-poor conditions. For Cd_P_ and Cd_O_, both of the two configurations will not be the relevant configurations for Cd doping in Ag_3_PO_4_, on account of the strong covalent interactions in the tetrahedral PO_4_ structural units. For interstitial Cd (Cd_i_), the calculated formation energy is relatively high compared with that of Cd_Ag_ donor. Our results demonstrate that Cd can bind to N atom to form a stable passive (Cd_Ag_-N_O_) complex. Although the transition energy level of the acceptor (Cd_Ag_-2N_O_) complex is reduced from 0.49 to −0.05 eV when electrons are transited from the impurity band of the passive complex rather than the original valence band, the formation energy of the acceptor complex is high in comparison with the isolated N_O_. Same as Cd_P_ and Cd_O_, Li_P_ and Li_O_ will not be the relevant configurations for Li doping in Ag_3_PO_4_. But interstitial Li (Li_i_ or Li_i_(s)) with suitable level and appreciable solubility is likely to be the *n*-type dopant under O-poor condition. For the (Li_Ag_-N_O_) complex, the calculated binding energy is negative (−0.11 eV) because the Ag-N bond is stronger than the Li-N bond. While, the (Li_Ag_-V_Ag_) complex is potentially to be the *p*-type dopant under O-rich condition.

## Additional Information

**How to cite this article**: Huang, Y. *et al*. Cadmium and lithium doping in silver orthophosphate: An *ab initio* study. *Sci. Rep.* **6**, 32574; doi: 10.1038/srep32574 (2016).

## Figures and Tables

**Table 1:** Calculated transition energy levels and binding energies *E*_b_ of the defect complexes, and defect formation energies with *E*_F_ = 0 under O-rich and O-poor conditions in Ag_3_PO_4_.

Cd							
Donor	ε(0/+)	ε(0/2+)	ε(0/3+)	ε(0/4+)	*E*_b_	Δ*H*_f_ (O-rich)	Δ*H*_f_ (O-poor)
Cd_Ag_	0.01					0.43	−0.42
Cd_o_	−0.02	−0.24	0.53	0.91		8.59	5.21
Cd_i_	0.27	0.14				4.74	3.05
Cd_i_(s)	0.1	0.01				4.90	3.21
**Acceptor**	**ε(−/0)**	**ε(2−/0)**	**ε(3−/0)**	**ε(4−/0)**	****Eb**	**Δ*H*_f_ (O-rich)**	**Δ*H*_f_ (O-poor)**
Cd_P_	0.13	0.27	0.25			7.89	10.42
Cd_Ag_-2N_O_	0.49				0.51	8.80	4.57
Cd_Ag_-2V_Ag_	0.18				−0.08		
N_O_	0.46					5.46	3.77
V_Ag_	0.18					0.46	1.35
**Li**							
**Donor**	**ε(0/+)**	**ε(0/2+)**	**ε(0/3+)**	**ε(0/4+)**	****Eb**	**Δ*H*_f_ (O-rich)**	**Δ*H*_f_ (O-poor)**
Li_O_	0.18	1.55	2.0			5.90	3.37
Li_i_	−0.04					2.22	1.38
Li_i_(s)	−0.15					2.16	1.32
**Acceptor**	**ε(−/0)**	**ε(2−/0)**	**ε(3−/0)**	**ε(4−/0)**	****E_b_**	**Δ*H*_f_ (O-rich)**	**Δ*H*_f_ (O-poor)**
Li_P_	0.70	0.69	0.85	0.89		6.62	10.0
Li_Ag_-N_O_	0.54				−0.11	3.51	1.82
Li_Ag_-V_Ag_	−0.04				0.04		

All energies are in eV.
